# Tailoring an educational program on the AHRQ Patient Safety Indicators to meet stakeholder needs: lessons learned in the VA

**DOI:** 10.1186/s12913-018-2904-5

**Published:** 2018-02-14

**Authors:** Marlena H. Shin, Peter E. Rivard, Michael Shwartz, Ann Borzecki, Enzo Yaksic, Kelly Stolzmann, Lisa Zubkoff, Amy K. Rosen

**Affiliations:** 1 Center for Healthcare Organization and Implementation Research (CHOIR), VA Boston Healthcare System, Boston, MA USA; 2 Sawyer Business School, Suffolk University, Boston, MA USA; 3 Questrom School of Business, Boston University, Boston, MA USA; 4 Center for Healthcare Organization and Implementation Research (CHOIR), Bedford VA Medical Center, Bedford, MA USA; 5 Department of Internal Medicine, Boston University School of Medicine, Boston, MA USA; 6 Department of Health Law, Policy and Management, Boston University School of Public Health, Boston, MA USA; 7 Massachusetts Veterans Epidemiology Research and Information Center, VA Boston Healthcare System, Boston, MA USA; 8 VA National Center for Patient Safety, Field Office, White River Junction, VT USA; 9 White River Junction VA Medical Center, White River Junction, VT USA; 10 Geisel School of Medicine, Dartmouth College, Hanover, NH USA; 11 Department of Surgery, Boston University School of Medicine, Boston, MA USA

**Keywords:** (3–10 words): Patient safety indicators, Patient safety, Quality indicators, Education, Program development, Implementation, Evaluation, Stakeholder engagement, Veterans health administration

## Abstract

**Background:**

Given that patient safety measures are increasingly used for public reporting and pay-for performance, it is important for stakeholders to understand how to use these measures for improvement. The Agency for Healthcare Research and Quality (AHRQ) Patient Safety Indicators (PSIs) are one particularly visible set of measures that are now used primarily for public reporting and pay-for-performance among both private sector and Veterans Health Administration (VA) hospitals. This trend generates a strong need for stakeholders to understand how to interpret and use the PSIs for quality improvement (QI). The goal of this study was to develop an educational program and tailor it to stakeholders’ needs. In this paper, we share what we learned from this program development process.

**Methods:**

Our study population included key VA stakeholders involved in reviewing performance reports and prioritizing and initiating quality/safety initiatives. A pre-program formative evaluation through telephone interviews and web-based surveys assessed stakeholders’ educational needs/interests. Findings from the formative evaluation led to development and implementation of a cyberseminar-based program, which we tailored to stakeholders’ needs/interests. A post-program survey evaluated program participants’ perceptions about the PSI educational program.

**Results:**

Interview data confirmed that the concepts we had developed for the interviews could be used for the survey. Survey results informed us on what program delivery mode and content topics were of high interest. Six cyberseminars were developed—three of which focused on two content areas that were noted of greatest interest: learning how to use PSIs for monitoring trends and understanding how to interpret PSIs. We also used snapshots of VA PSI reports so that participants could directly apply learnings. Although initial interest in the program was high, actual attendance was low. However, post-program survey results indicated that perceptions about the program were positive.

**Conclusions:**

Conducting a formative evaluation was a highly important process in program development. The useful information that we collected through the interviews and surveys allowed us to tailor the program to stakeholders’ needs and interests. Our experiences, particularly with the formative evaluation process, yielded valuable lessons that can guide others when developing and implementing similar educational programs.

**Electronic supplementary material:**

The online version of this article (10.1186/s12913-018-2904-5) contains supplementary material, which is available to authorized users.

## Background

Patient safety measures are increasingly used for public reporting and pay-for-performance [1–3]. Implicit in this use of the measures is the assumption that it will drive improvement, which continues to be critical [4]. Thus, it is important for the key stakeholders within organizations whose performance is being measured to understand and appropriately use some of these measures to identify, prioritize, and act on safety improvement opportunities [5–9]. One particularly visible set of measures in this domain is the Agency for Healthcare Research and Quality (AHRQ) Patient Safety Indicators (PSIs) [10, 11]. Originally developed for case finding activities and quality improvement (QI) [11, 12], the PSIs are now used primarily for public reporting and pay-for-performance among both private sector and Veterans Health Administration (VA) hospitals [1, 3, 13]. However, similar to other administrative data-based measures, PSIs can present particular challenges when being interpreted for QI purposes [5]. Stakeholders with inadequate understanding of the PSIs may also be averse to working with them for QI [5–9].

Among the strategies that have been used to educate users and other stakeholders about the PSIs are the AHRQ Quality Indicators Toolkit [14], a modified version of the Institute for Healthcare Improvement Virtual Breakthrough Series (IHI VBTS) [15], and AHRQ’s podcasts/cyberseminars [16]. Although the AHRQ Quality Indicators Toolkit and IHI VBTS have provided hospitals with an opportunity to learn about and implement PSI-related QI initiatives, these approaches can be very resource-intensive. On the other hand, similar to tele- and web-based training programs [17, 18], cyberseminars can be less resource intensive, and allow for widespread participation and dissemination of information to stakeholders [17, 18]. Evidence also suggests that cyberseminars can be a viable strategy for educating individals about clinical and research issues and findings [16, 18–20]. However, there is little empirical evidence as to the relative usefulness of these various strategies in facilitating stakeholder engagement and educating them about performance measures. To actively engage stakeholders in an educational program, it is important to tailor the program according to their needs and interests [21–23].

The present study was prompted by information within the VA that public reporting of VA PSI rates was imminent. Based on interviews with VA patient safety managers in a prior study [24], we knew that knowledge about the PSIs within the VA was both sparse and inconsistent. To address this gap, we obtained funding to develop an educational program that was tailored to stakeholders’ needs and could potentially help them in learning more about the PSIs for QI. Our study’s specific aims were to: 1) obtain VA stakeholders’ input on their educational needs related to the PSIs; 2) develop and implement a PSI educational program tailored to stakeholders’ needs; and 3) explore stakeholders’ perceptions about the program. The purpose of this paper is to share what we learned about developing a quality indicators education program (specifically, the AHRQ PSIs) and tailoring it to stakeholders’ needs and interests.

## Methods

### Overview

We conducted an implementation and evaluation study from 2011 to 2013. We began with a formative evaluation [25] involving telephone interviews and web-based surveys with stakeholders, which allowed us to learn more about their PSI educational needs and how the program should be tailored. Next, we developed and implemented a program based on the formative evaluation. Finally, we conducted a post-program evaluation to explore stakeholders’ perceptions about the program. We received approval from the Institutional Review Board (IRB) at the VA Boston Healthcare System (IRB #2563). Study participation was voluntary; we obtained informed consent from participants for each study aim.

### Study population

Our overall study population consisted of key VA stakeholders at 132 VA acute-care hospitals nationwide who were involved in quality/safety initiatives: middle managers (Quality/Performance Improvement Managers/Officers, and Patient Safety Managers/Officers) and senior managers (Medical Center Directors, Chiefs of Staff, Nurse Executives/Associate Directors of Patient Care Services, Associate Directors).

Within the overall population, we anticipated higher levels of participation from the middle manager group than the senior managers, based on our prior work [24]. The middle managers are most likely to be the initial recipients of performance reports, have a strong understanding of hospital safety and quality improvement programs and priorities, initiate improvement priorities based on performance reports, and be the first in line to provide training to other managers (senior, middle, and unit) within the organization about performance measures.

However, we did include senior managers because they are also key stakeholders: they are heavily involved in setting their organizations’ improvement priorities and reviewing performance reports with the patient safety, quality, and performance improvement managers for their hospital. Thus, when developing the PSI educational program, it was also important to understand their needs on learning about the PSIs.

### Formative evaluation: Telephone interviews

As a first step in the formative evaluation, we sought interviews with informants from eight of the 132 VA hospitals. We selected these sites based on their geographic diversity, variation in PSI rates, hospital size (number of Veterans served), and our knowledge about these facilities’ QI activities within the VA. Within each of these sites, we recruited 16 potential informants from our middle manager stakeholder group—all either Patient Safety Managers or Quality/Performance Improvement Managers—for the reasons described above, as hospital-level content experts on PSI-related learning needs at their facilities.

We developed an interview guide based on our experience from a prior PSI study and a literature review [24, 26–28]. This guide covered four a priori concepts: education about performance measures (“performance measure education”), education about the PSIs (“PSI education”), knowledge about the PSIs (“PSI knowledge”), and prioritization of improvement efforts within the organization (“improvement prioritization”). Additional file 1 provides the interview guide that we developed and used for the telephone interviews. We sought to obtain a general understanding of potential PSI educational needs and to assess whether similar a priori concepts should inform the survey questionnaire. Using the detailed notes taken during the 30-min telephone interviews, we summarized informants’ answers and conducted a rapid content analysis to identify evidence related to the a priori concepts and to identify emergent themes that would then guide development of the survey [29, 30].

### Formative evaluation: Pre-program web-based survey

Using the findings from the interviews, we developed a pre-program web-based survey that we then administered to all key VA stakeholders (described in the study population section above). The survey consisted of 56 closed-ended questions covering the a priori concepts plus the additional concepts that emerged from the data analysis of the interview guides as well as an additional literature review on organizational improvement [31]. Concepts included: awareness of performance reporting (“performance reporting”), stakeholders’ current/planned use of the PSIs (“PSI use”), and facilitators/barriers to PSI use and QI activities (“facilitators/barriers”). Response options included a five-point Likert scale (1 = strongly disagree to 5 = strongly agree) and yes/no. In addition, there were five open-ended questions where respondents could elaborate upon some of their answers. Additional file 2 provides the pre-program web-based survey that we administered to all key VA stakeholders.

The web-based survey was programmed and administered using Verint® Enterprise Feedback Management, a VA-approved software package that is widely used for survey development and administration. We used descriptive statistics to characterize our study sample and their responses to the survey questions, and qualitatively analyzed the open-ended responses to identify prevalent themes across respondents.

### Development and implementation of the PSI educational program

Program development was informed by the formative evaluation survey results. It was also informed by a review of existing patient safety/quality educational materials from organizations including VA Health Services Research and Development’s Center for Information Dissemination and Education Resources (CIDER), AHRQ, and IHI [14, 16, 20, 32, 33]. Once our PSI educational program was developed, we emailed all the VA stakeholders who had responded to the survey and invited them to participate in the educational program. We implemented the program over a four-month period (December 2012–March 2013).

### Post-program evaluation: Web-based survey

To learn about stakeholders’ perceptions of the program, a post-program evaluation survey was administered approximately 1 month after the PSI education program ended. This survey, consisting of 90 closed-ended questions and 12 open-ended questions, was administered to those VA stakeholders who had both (1) responded to the pre-program survey and (2) registered for the educational program. Response options were either a five-point Likert scale that ranged from 1 = strongly disagree to 5 = strongly agree, or yes/no. Additional file 3 provides the post-program web-based survey that we administered to stakeholders.

We used similar survey administration processes and data analyses for both the pre-program and post-program surveys. In addition, we matched the pre- and post-responses of individuals that participated in both surveys. Our analyses included comparison of pre- and post-program survey responses for the same 7 questions that appeared on both surveys.

## Results

### Obtain VA stakeholders’ input on their educational needs related to the PSIs.

#### Formative evaluation: Telephone interviews

We interviewed nine of the 16 potential informants: four Patient Safety Managers and five Quality/Performance Improvement Managers. This included at least one informant from each of the eight selected sites. Several themes that emerged from the interviews (below) helped guide our development of the formative evaluation pre-program survey and were also taken into consideration when developing the educational program:

- Some informants had heard about the PSIs at meetings held by national VA quality and safety offices, whereas other informants had not heard about the PSIs and were not aware that PSI rates were in reports that they received.
- Some were unclear on how to interpret PSIs.
- While some had received education on other performance measures from national VA offices, most reported that they had not received any formal education on the PSIs.
- Informants suggested ways they would like the PSI education to be delivered, such as web-based training and information placed on a VA SharePoint.
- Informants suggested making the educational program more relevant to the audience we were trying to educate and also more interactive.
- Informants believed that improvement priorities can be heavily driven by a hospital’s performance on quality/safety measures.
- Informants felt that improvement priorities are largely set by senior management according to internal hospital priorities; however, they can also be set according to mandates from the VA National and Regional Offices.

The interviews informed us about stakeholders’ potential PSI educational needs as well as aspects of organizational contextual factors that may drive hospital-level improvement priorities. Review of the interview data confirmed that the concepts we had developed for the interviews could be used for the survey. Because we were able to identify helpful information through our a priori concepts and interview questions, we used similar concepts and several of the specific interview questions in the survey questionnaire. For example, in the interview, we asked informants “If you were to receive education on the PSIs, how would you like it to be delivered?” Some of the options that emerged from informants’ answers to this question included: having access to materials on a website, conducting presentations of case studies, conducting presentations via LiveMeeting (a widely used VA web-based meeting platform), and including specific information about the PSIs in a handout or PowerPoint. We then included several of those options as response items when developing the survey question:

I would prefer to learn about the PSIs through:

- Web conferencing (e.g. LiveMeeting)
- Video conferencing (e.g., v-tel)
- Reports or journal articles
- Written case studies
- Video/audio materials (e.g., links to pre-recorded materials)
- Face-to-face conference
- Q&A sessions (e.g., via teleconference)

Table 1 provides additional examples of the concepts, interview questions, and survey questions that were developed based on the interviews.

**Table 1:** Examples of concept, interview question, and survey question

Concept	Interview question → Survey question
PSI Knowledge	Have you heard of the PSIs prior to this interview? → I am aware of the PSIs.
	Have you ever received a report that contains the PSI rates? → I currently receive reports containing rates for selected PSIs from Inpatient Evaluation Center (IPEC).
PSI Education	Have you received any education about the PSIs? → I have received education about the PSIs from VA Central Office [e.g., Inpatient Evaluation Center (IPEC), National Center for Patient Safety (NCPS)].
	If you were to receive education on the PSIs, how would you like it to be delivered? → I would prefer to learn about the PSIs through: web conferencing, video conferencing, reports or journal articles, written case studies, video/audio materials (e.g., links to pre-recorded materials), face-to-face conference, Q&A sessions (e.g., via teleconference).
Improvement Prioritization	Are priorities within your organization set in response to VACO or VISN mandates? → Patient safety/quality priorities for my facility are set mostly in response to: VACO mandates, VISN mandates, within the facility.
	Are priorities driven by performance measures vs. driven by other indicators (e.g., adverse events that occur, employee concerns, strategic planning, etc.)? → Patient safety/quality priorities at my facility are mostly driven by VACO performance measures. → Patient safety/quality priorities at my facility are mostly driven by other factors: adverse events that have occurred, employee concerns, strategic planning, press/media/public relations.

#### Formative evaluation: Web-based survey

We administered the pre-program survey to 782 VA stakeholders at 94 out of the 132 VA hospitals (71%); 181 (23%) responded. Table 2 shows the range of positions of these survey respondents. A majority of the respondents were Quality/Performance Improvement Managers/Officers and Patient Safety Managers/Officers. The surveys were helpful in informing us as to whether stakeholders understood the PSIs in the reports they received, and what their educational needs were related to the PSIs.

**Table 2:** Pre-program survey respondents (*n* = 181)

Position title	Number	Percent
Middle Managers		
Quality/Performance Improvement Manager/Officer	50	28
Patient Safety Manager/Officer	41	22
Senior Managers		
Medical Center Director	31	17
Associate Director for Nursing and Patient Care Services/ Chief Nurse Executive	27	15
Chief of Staff (i.e., Chief Clinical Officer)	17	10
Associate Medical Center Director	15	8
Total	181	100

**Fig. 1:**
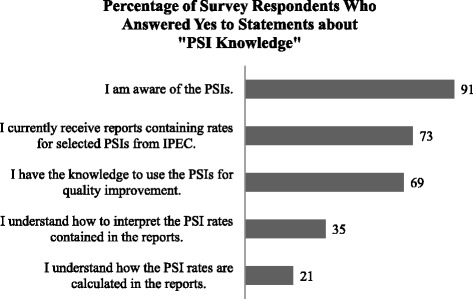
Pre-program survey responses to statements about “PSI Knowledge”

**Fig. 2:**
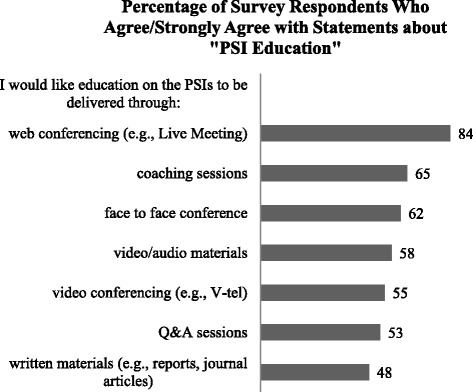
Pre-program survey responses to statements about “PSI Education”: delivery mode

**Fig. 3:**
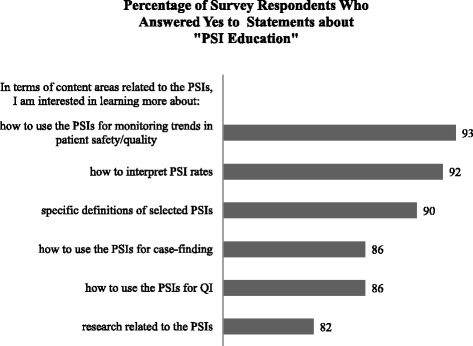
Pre-program survey responses to statements about “PSI Education”: content areas

The majority of respondents indicated that they were aware of the PSIs and had received reports containing PSI rates (91% and 73%, respectively), yet their understanding of how rates were calculated and interpreted was considerably lower (21% and 35%, respectively). Almost 50% of respondents reported having received education about the PSIs from VA Central Office. They were most interested in receiving the education via web-conferencing (e.g., LiveMeeting) (84%); education about the PSIs through written materials was of least interest. Respondents’ top two PSI-related educational interests were in monitoring trends in patient safety/quality (93%) and interpreting PSI rates (92%), although many of the other content areas also appeared to be of interest(> 80% of respondents answered yes to learning more about how to use the PSIs for case finding and QI, and research related to the PSIs). Figures 1, 2, and 3 provide additional details about survey responses for “PSI Knowledge,” “PSI Education” (delivery mode), and “PSI Education” (content areas), respectively.

**Fig. 4:**
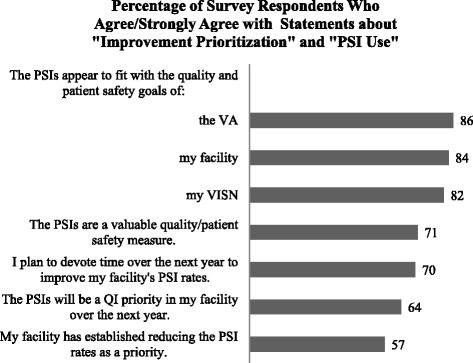
Pre-program survey responses to statements about “Improvement Prioritization” and “PSI Use”

In addition, we learned more about whether survey respondents perceived the PSIs as an organizational priority and what factors might be barriers to using the PSIs for QI. As shown in Fig. 4 (survey responses about “Improvement Prioritization” and “PSI Use”), while respondents agreed that the PSIs fit within the quality/patient safety goals of the VA (86%), were a valuable quality/safety measure (71%), and time would be devoted to improve rates (70%), there was less agreement on whether the PSIs were a current priority (57%) and would be an improvement priority at their hospital in the future (64%). In the open-ended response section, respondents most frequently noted the following as barriers to adopting the PSIs for patient safety/quality improvement initiatives: lack of training on the PSIs, lack of understanding and knowledge about the PSI data, lack of timeliness of VA performance reports (e.g., not real-time), and having too many measures and too much data to manage.

### Develop and implement a PSI educational program based on stakeholders’ input.

We invited all 181 VA stakeholders who responded to the pre-program survey to participate in the educational program; 82 (45%) registered to participate. Guided by stakeholders’ input from the findings of the pre-program survey and the interviews, we developed and implemented a PSI educational program that was tailored to stakeholders’ needs in the following ways:

#### Program delivery mode

- We offered six cyberseminar sessions via LiveMeeting (a web-conferencing platform used throughout the VA) because survey respondents preferred this mode. Each session was 45 min and was offered once. On average, nine participants attended each of the cyberseminar sessions. A majority of cyberseminar participants were from our middle manager stakeholder group.
- In addition, by offering the program as cyberseminars, we were able to easily record and archive the sessions on the program’s SharePoint site so that stakeholders who were unable to attend the live cyberseminars, or who wanted to revisit the sessions, could readily access them. (Recordings can be made available upon request from the corresponding author.) This allowed us to tailor the program based on stakeholders’ input from the interview.
- To make the sessions more interactive, the cyberseminars were conducted in an interview format, similar to the style that AHRQ uses in its podcasts about the Quality Indicators and Toolkit, with a moderator interviewing a speaker. Cyberseminar speakers were study team members with extensive knowledge about the topic presented, or representatives from our VA national partners’ offices [VA National Center for Patient Safety (NCPS) and VA Inpatient Evaluation Center (IPEC)]. Using LiveMeeting also facilitated an interactive platform, as it allowed participants to ask questions at any time during the presentation and receive answers directly from the speakers at that time.
- We integrated snapshots of the VA’s PSI report into our cyberseminars so that participants could directly apply what they learned from our program because survey respondents indicated that they were interested in learning how to use the PSIs, and interview informants suggested making the education more relevant to participants.

#### Program content

- Cyberseminar session topics and content corresponded to survey respondents’ expressed needs and interests. For example, because respondents indicated that they did not understand how to interpret the PSI rates in reports, we developed two sessions substantially devoted to this (Session 2: The PSIs and Your Facility’s Report; Session 3: How to Interpret PSI Rates). These sessions also focused on two of the three content areas that were of most interest to survey respondents (how to interpret PSI rates and specific definitions of selected PSIs). Table 3 provides additional information about content areas of interest as indicated by survey respondents, each cyberseminar session, topic, and outline of content covered.
- We also developed educational materials to complement what participants were learning in the cyberseminars: 1) an informational sheet, “Interpreting the AHRQ PSIs: A Basic Overview,” which was to be used, along with the cyberseminars, as a tool to help in interpreting and understanding the PSIs, as provided in Additional files 4 and 2) a matrix that highlighted information on each session and provided a list of materials referenced in each session for ease of access to materials, as provided in Additional file 5.

**Table 3:** Content area of interest for the PSI Educational Program as indicated by survey respondents: cyberseminar session, topic, and outline of information covered

Content Area of Interest for the PSI Educational Program (Survey Response %, Fig. 2)	Cyberseminar Session and Topic	Outline of Information Covered
	Session 1: An Overview of the PSI Educational Program	• Brief history about the study and the development of the PSI program • Road map of the cyberseminars and what topics will be covered during the cyberseminars
• How to interpret PSI rates (92%) • Specific definitions of selected PSIs (90%)	Session 2: The PSIs and Your Facility’s Reports	• Overview of the PSIs (e.g., definitions of PSIs) • Public reporting of the PSIs • Centers for Medicare and Medicaid Services Hospital Compare website for non-VA hospitals • VA reporting of the PSIs • Review of a sample VA PSI report • Where to find and request additional information on your facility’s VA PSI report • Who to contact with questions about your facility’s VA PSI report
• How to interpret PSI rates (92%) • Specific definitions of selected PSIs (90%)	Session 3: How to Interpret PSI Rates	• Further review of the PSIs (e.g., definitions of additional PSIs) • How PSIs are calculated (e.g., observed rates vs. risk adjusted rates) • How valid are the PSIs • Positive predictive values • Reasons for false positive findings • Negative predictive values • Processes of care
• How to use the PSIs for monitoring trends (93%) • How to use the PSIs for case-finding (86%)	Session 4: How to Use the PSIs and Organizational Factors to Consider	• AHRQ Quality Indicators Toolkit • Monitoring trends/benchmarking/case finding • Identifying priorities for PSI rate improvement • Organizational factors to consider
• How to use the PSIs for QI (86%)	Session 5: Using the PSIs for QI: Experiences Within and Outside the VA	• Using the PSIs for quality improvement • IHI Virtual Breakthrough Series on Postoperative Respiratory Failure • AHRQ Quality Indicators Toolkit • Case study from AHRQ newsletters
	Session 6: Wrap up call/Q&A session	• Summary of what cyberseminars covered • Q&A session

### Explore stakeholders’ perceptions about the program.

#### Post-program evaluation: Web-based survey

Thirteen out of the 82 stakeholders who had registered to attend the cyberseminars responded to both the pre- and post-program surveys. In general, respondents’ answers between pre- and post-program survey questions reflected positive changes for “PSI knowledge,” although changes were somewhat negative for “PSI use.” Table 4 provides the findings of those 7 items that were asked in both the pre- and post-program surveys. For example, we found that for the two questions about “PSI Knowledge” (I understand how to interpret the PSI rates contained in the reports; and I understand how PSI rates are calculated in the reports), survey respondents perceived that they better understood how to interpret and understand the PSI rates after our program, as noted by the positive changes between pre- and post-program surveys.

**Table 4:** Changes between pre- and post-program survey responses (*N* = 13)

Question	Response		Number of Respondents	% Positive Change*	% Negative Change^
	Pre	Post			
I have received education about the PSIs from VA Central Office (e.g., IPEC, NCPS).	Yes	Yes	4	3/13 = 23%	1/13 = 8%
	No	Yes	2*		
	No response	Yes	1*		
	No	No	5		
	Yes	No	1^		
I understand how the PSI rates are calculated in reports.	Yes	Yes	2	8/13 = 62%	2/13 = 15%
	No	Yes	4*		
	No response	Yes	4*		
	No	No	1		
	No response	No	2^		
I understand how to interpret the PSI rates contained in reports.	Yes	Yes	2	8/13 = 62%	2/13 = 15%
	No	Yes	4*		
	No response	Yes	4*		
	No	No	1		
	No response	No	2^		
My facility has established reducing PSI rates as a priority.	Strongly agree	Agree	2^	1/13 = 8%	4/13 = 31%
	Agree	Strongly agree	1*		
	Agree	Agree	2		
	Agree	Disagree	1^		
	Neither agree nor disagree	Neither agree nor disagree	3		
	Neither agree nor disagree	No response	1^		
	Disagree	Disagree	2		
	No response	No response	1		
The PSIs are a valuable quality/patient safety measure.	Strongly agree	No response	1^	1/13 = 8%	6/13 = 46%
	Agree	Strongly agree	1*		
	Agree	Agree	5		
	Agree	Neither agree nor disagree	2^		
	Agree	No response	1^		
	Neither agree nor disagree	Neither agree nor disagree	1		
	Neither agree nor disagree	No response	2^		
The PSIs will be a QI priority in my facility over the next year.	Strongly agree	Strongly agree	1	1/13 = 8%	5/13 = 38%
	Agree	Agree	3		
	Agree	Neither agree nor disagree	1^		
	Agree	Disagree	1^		
	Neither agree nor disagree	Neither agree nor disagree	2		
	Neither agree nor disagree	Disagree	1^		
	Neither agree nor disagree	No response	2^		
	No response	Agree	1*		
	No response	No response	1		
I plan to devote time over the next year to improve my facility’s PSI rates.	Strongly agree	Agree	1^	2/13 = 15%	5/13 = 39%
	Agree	Agree	2		
	Agree	Neither agree nor disagree	2^		
	Neither agree nor disagree	Neither agree nor disagree	3		
	Neither agree nor disagree	Agree	1*		
	Neither agree nor disagree	No response	2^		
	No response	Agree	1*		
	No response	No response	1		

Overall, survey respondents had positive perceptions of the PSI educational program. A majority of respondents agreed/strongly agreed that the sessions were useful (73%), were satisfied with what they learned (64%), and would recommend this program to their staff/colleagues (82%). Sessions 2 (“The PSIs and Your Facility’s Reports”) and 3 (“How to Interpret PSI Rates”) contributed most to respondents’ learning about the PSIs.

Respondents noted several factors in the open-ended questions that contributed the most to their learning about the PSIs, including: the program’s structure (e.g., LiveMeeting, archived/recorded sessions on study SharePoint site to access on their own time, availability of archived handouts) and content (e.g., clear, concise, and then applied examples, how VA obtains the data to calculate the PSI rates, what the PSIs measure and their limitations). Respondents noted that PSI data validity concerns were a barrier to using the PSIs for QI at their hospitals (e.g., coding issues and clinical staff will not “believe the PSI rates”).

## Discussion

We conducted this implementation and evaluation study with the goal of developing an educational program that was tailored to stakeholders’ needs. Ultimately, we hoped that our educational program would help VA stakeholders learn more about how to interpret and use the PSIs for QI. Our experiences in developing, implementing, and evaluating a PSI educational program yielded several valuable lessons.

First, consistent with the literature [21–23, 25, 34–36], we found that stakeholder input was highly useful for program development, emphasizing the importance of conducting a formative evaluation with the key stakeholders involved in the specific activities to which the program is most relevant. Through our formative evaluation, we engaged stakeholders in the development process, and built and tailored a program based on their needs and interests. Interviews with a small sample of hospital-level content experts were adequate to provide us with a general awareness of the potential needs and preferences of the larger stakeholder population. This helped us set the scope of the stakeholder survey in a way that yielded very informative results.

Second, the formative evaluation enabled us to tailor the program to stakeholders’ needs and preferences. In particular, the formative evaluation influenced two key components of our educational program: the content areas for the cyberseminars and the mechanism by which the content should be delivered. Although the impact of the program was limited by a low participation rate, our post-program survey results showed that those respondents who did participate expressed positive perceptions of what they had learned from the program.

Third, we learned that the timing of program implementation can be critical for success. When we began the study, we had anticipated that public reporting of VA PSI rates was imminent. However, as we moved forward with implementation, public reporting of VA PSI rates was postponed. Similar to what we found, prior studies show that hospitals’ responses to public reporting of performance measures may differ depending on hospitals’ perceptions of the need to improve on a given measure [7–9]. Public reporting of a performance measure may provide the urgency and impetus for a hospital to act on reports of poor performance [7–9]. In our study, postponement of public reporting of the PSIs apparently mitigated this sense of urgency as well as impetus for hospitals to seek active guidance on ways to improve PSI rates. This shift in timing may partly explain why attendance at our cyberseminars and response rates to our post-program surveys were relatively low, despite the initial high interest in our program. This may also explain why survey respondents’ perceptions about PSI use may have changed. Given that, as this is written, we are seeing increased interest in PSIs due to reporting on the Centers for Medicare and Medicaid Services (CMS) Hospital Compare and the VA Strategic Analytics for Improvement Learning (SAIL), we presume that VA stakeholders would now be much more receptive to education on how to interpret and use the PSIs. Our study emphasizes the importance of aligning program implementation with organizational priorities to ensure proper timing, active engagement by stakeholders, and stronger buy-in from key leadership.

Fourth, this study adds to the limited empirical evidence in the literature about the most useful strategies on how to educate stakeholders about the PSIs. Respondents preferred to obtain PSI education through web-based meetings (e.g., cyberseminars). Consistent with the literature, we also learned that cyberseminars were relatively easy to implement, could be used to widely disseminate information, and appeared to be less resource-intensive than other options [19]. Our findings suggest that cyberseminars can, indeed, be a suitable strategy to educate stakeholders about the PSIs as well as other performance measures.

### Limitations and strengths

Our study has some limitations. As mentioned in the discussion above, although the program was developed through stakeholder input and initial interest in our PSI education program was high, actual attendance at our cyberseminar sessions was low. The response rate for our post-program survey was also low, potentially due to the timing of PSI reporting. In addition, although we intended the pre- and post-program surveys to take less than 20 min to complete, some respondents may have found the length of the surveys to be somewhat burdensome; thus, this may have impacted the response rate. Finally, while the U.S. is seeing growth in large integrated healthcare delivery systems that resemble the VA in some respects, there are, of course, organizational differences between the VA and those private sector systems.

However, our study had strengths worth highlighting. A major strength of the study is that we engaged stakeholders in developing the cyberseminars within a national healthcare system through interviews and surveys. As we learned from this study, while there are challenges in developing and implementing programs within a national healthcare system, the lessons learned allowed us to gain a better understanding of how different strategies can be useful for disseminating information about quality and safety measures and helping stakeholders to effectively use these measures. Despite the small sample size at the end, the PSI educational program was developed through wider stakeholder input, and had initial interest from numerous stakeholders. In addition, although our study findings are within the context of the VA setting and are focused on the PSIs (one performance measure), we have attempted to present the lessons learned in this study in ways that facilitate their application to other health care settings, other program (tool, strategy, intervention) development and implementation efforts, and other performance measures.

## Conclusions

Although this study focused on the AHRQ PSIs, our experiences yielded valuable lessons that others should consider when developing and implementing programs. Timing of implementation and alignment with organizational priorities are important elements for successful engagement of stakeholders in program development and implementation. However, we learned that one of the most critical elements of program development is the formative evaluation process, which we conducted through interviews and surveys. While the interviews provided us with an opportunity to engage with and get feedback from a smaller group of VA stakeholders (which helped to guide our development of the survey and gave us initial insight into educational needs), the pre-program survey allowed us to gather information more broadly across a range of VA stakeholders. Ultimately, through the formative evaluation process, we developed and implemented a PSI educational program that was tailored to stakeholders’ needs and interests by triangulating the information gathered from both methods.

## Additional files

Additional file 1: Formative Evaluation: Interview Guide. This file provides the interview guide we used for the telephone interviews to obtain a general understanding of potential PSI educational needs and assess whether similar a priori concepts should inform the survey. (PDF 168 kb)

Additional file 2: Formative Evaluation: Pre-program Survey. This file provides the survey that we administered to obtain stakeholders’ input on their educational needs related to the PSIs. (PDF 169 kb)

Additional file 3: Post-program Evaluation Survey. This file provides the post-program survey that we administered to learn about stakeholders’ perceptions of the PSI Educational Program. (PDF 259 kb)

Additional file 4: Informational Sheet, Interpreting the AHRQ PSIs: A Basic Overview. This file provides the informational sheet which PSI Educational Program participants could use to help them interpret and understand the PSIs. (PDF 87 kb)

Additional file 5: PSI Educational Program Matrix. This file highlights information covered in each session of the PSI Educational Program and provides a list of materials referenced in each session. (PDF 213 kb)

## Abbreviations

AHRQ: Agency for Healthcare Research and Quality
CIDER: Center for Information Dissemination and Education Resources
CMS: Centers for Medicare and Medicaid Services
IHI VBTS: Institute for Healthcare Improvement Virtual Breakthrough Series
IRB: Institutional Review Board
PSIs: Patient Safety Indicators
QI: Quality improvement
SAIL: Strategic Analytics for Improvement and Learning
VA: Veterans Health Administration
