# Supplementary material for: Tailoring an educational program on the AHRQ Patient Safety Indicators to meet stakeholder needs: lessons learned in the VA
Source: BMC Health Serv Res. 2018 Feb 14;18:114. doi: 10.1186/s12913-018-2904-5 (PMC5813330; doi:10.1186/s12913-018-2904-5)
Supplement: Supplementary file 1 — Formative Evaluation: Interview Guide. This file provides the interview guide we used for the telephone interviews to obtain a general understanding of potential PSI educational needs and assess whether similar a priori concepts should inform the survey. (PDF 168 kb) [file 12913_2018_2904_MOESM1_ESM.pdf]

Formative Evaluation: Interview Guide. This file provides the interview guide we used for the telephone interviews to obtain a general understanding of potential PSI educational needs and assess whether similar a priori concepts should inform the survey.

Background

1. Very briefly, what is your current position at this facility? How long have you been in this position?

CONCEPT: EDUCATION ABOUT PERFORMANCE MEASURES

VA

As I mentioned before, we're interested in getting some feedback about your educational needs based on the programs that you may have participated in previously.

2. In general, when VACO rolls out a new performance measure on LinKS/ASPIRE/Hospital Compare, what types of education do they provide to the field, if any, to introduce you to this measure (e.g., cyber seminars, written materials, Q&A session, etc.)?

3. Typically, which VA offices provide education to the field when new performance measures are rolled out?

4. Have you participated in any of the educational programs hosted by VACO about a specific performance measure(s)? If so, tell us about-
   Possible probes:
   a. For which performance measure(s)
   b. How the education was delivered (e.g., face-to-face, cyber seminar, internet, Q&A, discussion, Socratic, interactive/role playing, skits, collaborative)
   c. what was the structure of this education (e.g., format, time duration, number of participants-nationwide or VISN or facility specific, target participant group-PSMs, QMs, executive leaders)
   d. what you liked/did not like about this program (repeat questions a-e if multiple programs)
   e. if you have participated in multiple educational programs, which program did you find more effective/less effective and why

5. Now that we've discussed how VACO educates the field and the programs you have participated in, let's briefly discuss what happens at your facility. When VACO rolls out a new performance measure on LinKS/ASPIRE/Hospital Compare, what action does your organization typically take? (e.g., provide education about this measure?)
   Possible probes:
   a. If education was provided to staff, how was the education delivered and to whom?
   b. How was the decision made as to who would participate in this education?

<u>Non-VA (e.g., IHI, AHRQ)</u>

Now, I'd like to talk to you about any educational programs on performance measures that you have previously participated in through non-VA organizations, such as IHI or AHRQ.

6. Have you participated in any educational programs on performance measures hosted by non-VA organizations (e.g., IHI, AHRQ)? If so, tell us about-
   Possible probes:
   a. For which performance measure(s)
   b. Who hosted the program
   c. how the education was delivered (e.g., face-to-face, cyber seminar, internet, Q&A, discussion, Socratic, interactive/role playing, skits)
   d. what was the structure of this education (e.g., format, time duration, number of participants-nationwide or VISN, target participant group-PSMs, QMs, executive leaders)
   e. what you liked/did not like about this program (repeat questions a-e if multiple programs)
   f. if you have participated in multiple educational programs, which program did you find more effective/less effective and why

CONCEPT: KNOWLEDGE ABOUT THE PSIs

Now, I'd like to talk to you about the PSIs—whether you've attend any educational programs related to the PSIs and what you think might be a good educational program to develop in terms of structure and delivery method.

7. Have you heard of the PSIs prior to this interview?

8. Have you ever received a report that contains the PSI rates (your facility's PSI rates)? (e.g., LinKS)
   Possible probes:
   a. What information was contained in the report? (rates + any background information/explanation?)
   b. Did you find the information about the PSIs in the report understandable?
   c. Are there any ways in which it could have been improved?

CONCEPT: EDUCATION ABOUT THE PSIs

9. Have you received any education about the PSIs?
   Possible probes:
   a. Were they within the VA or outside the VA?
   b. Tell us about---
      i. Who hosted the program

ii. How the education was delivered (e.g., face-to-face, cyber seminar, internet, Q&A, discussion, Socratic, interactive/role playing, skits)
iii. What was the structure of this education (e.g., format, time duration, number of participants-nationwide or VISN, target participant group-PSMs, QMs, executive leaders)
iv. What you liked/did not like about this program (repeat questions a-e if multiple programs)
v. If you have participated in multiple educational programs about the PSIs, which program did you find more effective/less effective and why

10. If you were to receive education on the PSIs, how would you like it to be delivered?
    Possible probes:
    a. Internet-based (e.g., cyber seminars, listserv, FAQs)
    b. Discussion-based (e.g., Q&A sessions, learning from others' experiences)
    c. Resource-based (e.g., distribution of articles and other written materials)
    d. Combination-if so, what?
    e. Other-if so, what?

CONCEPT: IMPROVEMENT PRIORITIZATION WITHIN THE ORGANIZATION

Now that we've discussed your experiences with various educational programs and your preferences, we're interested in knowing how improvement priorities are set for your organization.

11. Are priorities within your organization set in response to VACO or VISN mandates?
    Possible probe:
    a. Most, half, little, none

12. Are priorities driven by performance measures vs. driven by other indicators (e.g., adverse events that occur, employee concerns, strategic planning, etc.)

13. Typically, which decision makers within your facility are involved in setting improvement priorities?
