# Supplementary material for: Tailoring an educational program on the AHRQ Patient Safety Indicators to meet stakeholder needs: lessons learned in the VA
Source: BMC Health Serv Res. 2018 Feb 14;18:114. doi: 10.1186/s12913-018-2904-5 (PMC5813330; doi:10.1186/s12913-018-2904-5)
Supplement: Supplementary file 2 — Formative Evaluation: Pre-program Survey. This file provides the survey that we administered to obtain stakeholders’ input on their educational needs related to the PSIs. (PDF 169 kb) [file 12913_2018_2904_MOESM2_ESM.pdf]

Formative Evaluation: Pre-program Survey. This file provides the survey that we administered to obtain stakeholders' input on their educational needs related to the PSIs.

The following questions are about the Centers for Medicare and Medicaid Services (CMS) and VA Hospital Compare websites.

Please provide a yes or no response to each of the following statements. If you prefer not to answer the question, please click "Decline to Respond."

**CONCEPT: PERFORMANCE REPORTING**	Yes	No	Decline to Respond
1. I am aware of the CMS Hospital Compare website.	1	2	3
2. I am aware of the VA Hospital Compare website.	1	2	3
3. I am aware of the mortality and/or readmissions outcomes measures on the CMS Hospital Compare website.	1	2	3
4. I am aware of the mortality and/or readmissions outcomes measures on the VA Hospital Compare website.	1	2	3

Please indicate your level of agreement or disagreement with each of the following statements.

Base your responses on your experience **over the past year**. If you prefer not to answer a question, please click "Decline to Respond."

CONCEPT: IMPROVEMENT PRIORITIZATION	Strongly Disagree	Disagree	Neither Agree nor Disagree	Agree	Strongly Agree	Decline to Respond
5. My facility has established reducing mortality as a priority.	1	2	3	4	5	6
6. My facility has established reducing readmissions as a priority.	1	2	3	4	5	6

The following questions are about the Patient Safety Indicators (PSIs).

Please provide a yes or no response to each of the following statements. If you prefer not to answer a question, please click "Decline to Respond." If you do not know the answers to the statements, please click "Do Not Know."

CONCEPT: PSI KNOWLEDGE	Yes	No	Decline to Respond
7. I am aware of the PSIs.	1	2	3

CONCEPT: PSI EDUCATION	Yes	No	Do Not Know	Decline to Respond
8. I have received education about the PSIs from VA Central Office [e.g., Inpatient Evaluation Center (IPEC), National Center for Patient Safety (NCPS)].	1	2	3	4

CONCEPT: PSI KNOWLEDGE	Yes	No	Do Not Know	Decline to Respond

9. I currently receive reports containing rates for selected PSIs from IPEC.	1	2	3	4

The following are additional questions about the PSIs.

You responded that you have received reports containing rates for selected PSIs from IPEC.

Please provide a yes, somewhat, or no response to each of the following statements. If you prefer not to answer a question, please click "Decline to Respond."

**CONCEPT: PSI KNOWLEDGE**	Yes	No	Do Not Know	Decline to Respond
10. I understand how the PSI rates are calculated in the reports.	1	2	3	4
11. I understand how to interpret the PSI rates contained in the reports.	1	2	3	4

The following are additional questions about the PSIs.

Please indicate your level of agreement or disagreement with each of the following statements. Base your responses on your experience **over the past year**. If you prefer not to answer a question, please click "Decline to Respond."

**CONCEPT: PSI KNOWLEDGE**	Strongly Disagree	Disagree	Neither Agree nor Disagree	Agree	Strongly Agree	Decline to Respond
12. I have the knowledge to use the PSIs for quality improvement (QI).	1	2	3	4	5	6

CONCEPT: PSI USE	Strongly Disagree	Disagree	Neither Agree nor Disagree	Agree	Strongly Agree	Decline to Respond
13. My facility currently uses the PSIs for QI.	1	2	3	4	5	6
14. My facility has established reducing PSI rates as a priority.	1	2	3	4	5	6
15. The PSIs are a valuable quality/patient safety measure.	1	2	3	4	5	6

CONCEPT: IMPROVEMENT PRIORITIZATION	Strongly Disagree	Disagree	Neither Agree nor Disagree	Agree	Strongly Agree	Decline to Respond
16. The PSIs appear to fit with the quality/patient safety goals of: The VA	1	2	3	4	5	6
17. The PSIs appear to fit with the quality/patient safety goals of: The VISN	1	2	3	4	5	6
18. The PSIs appear to fit with the quality/patient safety goals of: My facility	1	2	3	4	5	6

The following are additional questions about the PSIs.

Please indicate your level of agreement or disagreement with each of the following statements. Base your responses on what your experience will be **over the next year**. If you prefer not to answer a question, please click "Decline to Respond."

CONCEPT: PSI USE	Strongly Disagree	Disagree	Neither Agree nor	Agree	Strongly Agree	Decline to

			Disagree			Respond
19. The PSIs will be a QI priority in my facility over the next year.	1	2	3	4	5	6
20. I plan to devote time over the next year to improve my facility's PSI rates.	1	2	3	4	5	6

The following set of statements are about potential obstacles to adopting the PSIs at your facility.

Please indicate your level of agreement or disagreement with each of the following statements. Base your responses on your experience **over the past year**. If you prefer not to answer a question, please click "Decline to Respond."

There may be obstacles to adopting the PSIs for patient safety/quality improvement initiatives, such as **lack of**:

**CONCEPT: FACILITATORS/BARRIERS**	Strongly Disagree	Disagree	Neither Agree nor Disagree	Agree	Strongly Agree	Decline to Respond
21. Desire/impetus to change (e.g., no external pressure to incorporate PSI related work into my facility's QI projects)	1	2	3	4	5	6
22. Leadership commitment to incorporate the PSIs into my facility's QI work	1	2	3	4	5	6
23. Collaboration across divisions and disciplines	1	2	3	4	5	6
24. Staff engagement in improvement projects	1	2	3	4	5	6
25. Resources (e.g., human or fiscal)	1	2	3	4	5	6

If there are any other obstacles to adopting the PSIs for patient safety/quality improvement initiatives, please list them below. Q10A-Q10E

The following are questions about your educational interests and needs related to the PSIs.

Please indicate your level of agreement or disagreement with each of the following statements. If you prefer not to answer a question, please click "Decline to Respond."

**CONCEPT: PSI EDUCATION**	Strongly Disagree	Disagree	Neither Agree nor Disagree	Agree	Strongly Agree	Decline to Respond
26. I am interested in learning more about the PSIs.	1	2	3	4	5	6

In terms of content areas related to the PSIs, I am interested in learning more about:

**CONCEPT: PSI EDUCATION**	Yes	No	Decline to Respond
27. Specific definitions of selected PSIs	1	2	3
28. How to interpret PSI rates	1	2	3
29. How to use the PSIs for monitoring trends in patient safety/quality	1	2	3
30. How to use the PSIs for QI	1	2	3
31. How to use the PSIs for case-finding	1	2	3
32. Research related to the PSIs	1	2	3

If there are any other content areas related to the PSIs that you are interested in learning more about, please list them below. Q13A-Q13E

The following are additional questions about your educational interests and needs related to the PSIs.

Please indicate your level of agreement or disagreement with each of the following statements. If you prefer not to answer a question, please click "Decline to Respond."

I would prefer to learn about the PSIs through:

**CONCEPT: PSI EDUCATION**	Strongly Disagree	Disagree	Neither Agree nor Disagree	Agree	Strongly Agree	Decline to Respond
33. Web conferencing (e.g., LiveMeeting)	1	2	3	4	5	6
34. Video conferencing (e.g., v-tel)	1	2	3	4	5	6
35. Reports or journal articles	1	2	3	4	5	6
36. Written case studies	1	2	3	4	5	6
37. Video/Audio materials (e.g. links to pre-recorded materials)	1	2	3	4	5	6

38. Face to face conference	1	2	3	4	5	6
39. Q&A sessions (e.g. via teleconference)	1	2	3	4	5	6

If there are any other ways that you would prefer to learn about the PSIs, please list them below.
Q15A-Q15E

The following are additional questions about your educational interests and needs related to the PSIs.

Please indicate your level of agreement or disagreement with each of the following statements. If you prefer not to answer a question, please click "Decline to Respond."

For the methods previously checked in the last question, I would prefer to be educated with:

**CONCEPT: PSI EDUCATION**	Strongly Disagree	Disagree	Neither Agree nor Disagree	Agree	Strongly Agree	Decline to Respond
40. All VA facilities (nationwide)	1	2	3	4	5	6
41. Other facilities in my VISN	1	2	3	4	5	6

42. Other individuals from my facility	1	2	3	4	5	6

If there are any other groups you would prefer to be educated with, please list them below. Q17A-Q17E

The following questions return to the topic of how your facility sets patient safety/quality priorities.

Please indicate your level of agreement or disagreement with each of the following statements. Base your responses on your experience **over the past year**. If you prefer not to answer a question, please click "Decline to Respond."

**CONCEPT: IMPROVEMENT PRIORITIZATION**	Strongly Disagree	Disagree	Neither Agree nor Disagree	Agree	Strongly Agree	Decline to Respond
43. Patient safety/quality priorities for my facility are set mostly in response to VA Central Office (VACO) mandates.	1	2	3	4	5	6
44. Patient safety/quality priorities for my facility are set mostly in response to VISN mandates.	1	2	3	4	5	6
45. Patient safety/quality priorities for my facility are decided upon mostly within the facility.	1	2	3	4	5	6

The following are questions about how your facility sets patient safety/quality priorities to the PSIs.

Please indicate your level of agreement or disagreement with each of the following statements. Base your responses on your experience **over the past year**. If you prefer not to answer a question, please click "Decline to Respond."

**CONCEPT: IMPROVEMENT PRIORITIZATION**	Strongly Disagree	Disagree	Neither Agree nor Disagree	Agree	Strongly Agree	Decline to Respond
46. Patient safety/quality priorities at my facility are mostly driven by VACO performance measures.	1	2	3	4	5	6
47. Patient safety/quality priorities at my facility are mostly driven by Joint Commission performance measures.	1	2	3	4	5	6

Patient safety/quality priorities at my facility are mostly driven by other factors:

**CONCEPT: IMPROVEMENT PRIORITIZATION**	Strongly Disagree	Disagree	Neither Agree nor Disagree	Agree	Strongly Agree	Decline to Respond
48. Adverse events that have occurred	1	2	3	4	5	6
49. Employee concerns	1	2	3	4	5	6
50. Strategic planning	1	2	3	4	5	6
51. Press/media/public relations	1	2	3	4	5	6

If there are any other factors that drive patient safety/quality priorities at your facility, please list them below. Q21A-Q21E

The following are questions related to your facility.

Please indicate your level of agreement or disagreement with each of the following statements. Base your responses on your experience **over the past year**. If you prefer not to answer a question, please click "Decline to Respond."

**CONCEPT: FACLITATORS/BARRIERS**	Strongly Disagree	Disagree	Neither Agree nor Disagree	Agree	Strongly Agree	Decline to respond
52. Improvements in patient safety/quality measures do not occur at the rate I hope they would occur at my facility.	1	2	3	4	5	6
53. My facility is committed to providing additional resources, if required, over the next year to address improvements on quality/safety measures.	1	2	3	4	5	6
54. Most staff are involved in quality improvement and patient safety initiatives.	1	2	3	4	5	6
55. It has been difficult for front-line staff to be consistently involved in safety/quality improvement given all the demands on their time.	1	2	3	4	5	6
56. I am personally involved in efforts to improve quality or safety measures.	1	2	3	4	5	6
