# Supplementary material for: Tailoring an educational program on the AHRQ Patient Safety Indicators to meet stakeholder needs: lessons learned in the VA
Source: BMC Health Serv Res. 2018 Feb 14;18:114. doi: 10.1186/s12913-018-2904-5 (PMC5813330; doi:10.1186/s12913-018-2904-5)
Supplement: Supplementary file 3 — Post-program Evaluation Survey. This file provides the post-program survey that we administered to learn about stakeholders’ perceptions of the PSI Educational Program. (PDF 259 kb) [file 12913_2018_2904_MOESM3_ESM.pdf]

Post-program Evaluation Survey. This file provides the post-program survey that we administered to learn about stakeholders' perceptions of the PSI Educational Program.

Please provide a yes or no response to each of the following statements. If you prefer not to answer the question, please click "Decline to Respond."

	Yes	No	Do Not Know	Decline to Respond
1. Since June 2012, I have received education about the PSIs from VA Central Office (e.g., IPEC, National Center for Patient Safety). Note: When answering this question, please keep in mind that this does not include the education received through this study's PSI program.				

	Yes	No	Decline to respond
2. I understand how the PSI rates are calculated in the LinKS reports.			
3. I understand how to interpret the PSI rates contained in the LinKS reports.			

The following questions are about the study's PSI Educational Program and your opinions on the PSIs.

Please indicate your level of agreement or disagreement with each of the following statements. Base your responses on your experience with the PSI educational program. If you prefer not to answer a question, please click "Decline to Respond." Please answer the following questions if you viewed any of the program materials (i.e., listened to the presentation via Live Meeting, listened to the

presentation via archived recordings, reviewed PowerPoint slides on program's SharePoint site, or reviewed other materials, such as articles, weblinks, on the program's SharePoint site).

	Strongly Disagree	Disagree	Neither Agree nor Disagree	Agree	Strongly Agree	Decline to Respond
4. Overall, I am satisfied with what I learned through the program.	1	2	3	4	5	
5. Overall, I thought the session topics chosen for the program were useful.	1	2	3	4	5	
6. I would recommend this program to my staff/colleagues.	1	2	3	4	5	
7. The program was valuable in helping me learn about the PSIs.	1	2	3	4	5	
8. After reviewing the program materials, I feel competent to pass on knowledge about the PSIs to my staff/colleagues.	1	2	3	4	5	

	Strongly Disagree	Disagree	Neither Agree nor Disagree	Agree	Strongly Agree	Decline to Respond
9. I plan to disseminate the information learned from the program to my staff/colleagues.	1	2	3	4	5	
10. The knowledge gained from the program will be put to direct use in developing QI initiatives related to safety.	1	2	3	4	5	
11. I have the knowledge to use the PSIs for QI.	1	2	3	4	5	
12. My facility currently uses the PSIs for QI.	1	2	3	4	5	
13. My facility has established reducing PSI rates as a priority.	1	2	3	4	5	
14. The PSIs are a valuable quality/patient safety measure.	1	2	3	4	5	

	Strongly Disagree	Disagree	Neither Agree nor Disagree	Agree	Strongly Agree	Decline to Respond
15. The PSIs will be a QI priority in my facility over the next year.	1	2	3	4	5	
16. I plan to devote time over the next year to improve my facility's PSI rates.	1	2	3	4	5	
17. If the VA PSI rates were publicly reported on the VA and/or CMS Hospital Compare websites, they would more likely be used for QI activities by my facility.	1	2	3	4	5	
18. If the VA PSI rates were publicly reported on the VA and/or CMS Hospital Compare websites, I would pay more attention to the PSI rates on my LinKS report than I currently do.	1	2	3	4	5	

About the individual sessions:

The following questions are about the program's individual sessions.

Please indicate your level of agreement or disagreement with each of the following statements. Base your responses on your experience with the program's individual sessions. If you prefer not to answer a question, please click "Decline to Respond." Please answer the following questions if you viewed any materials from at least one of the sessions.

Session 1: An Overview of the PSI Educational Program (Shin)

19. Did you view or listen to any of the content for Session 1 (e.g., listened to the presentation via Live Meeting, listened to the presentation via archived recordings, reviewed the PowerPoint slide on Program's SharePoint site)?	Yes	No	Decline to Respond
**If yes or decline to respond, then ask Q2. If no, then skip to Session 2.**			
20. Did you listen to the presentation for Session 1?	Yes	No	Decline to Respond
**If yes or decline to respond, then ask Q2a and Q2b. If no, then skip to Q3.**			
21. Did you listen to the presentation via Live Meeting?	Yes	No	Decline to Respond
22. Did you listen to the presentation via the archived recording?	Yes	No	Decline to Respond
23. Did you review the PowerPoint slides on the VA SharePoint site for Session 1?	Yes	No	Decline to Respond

Session 2: The PSIs and Your Facility's Reports (Rosen and IPEC)

24. Did you view or listen to any of the content for Session 2 (e.g., listened to the presentation via Live Meeting, listened to the presentation via archived recordings, reviewed the PowerPoint slide on Program's SharePoint site)?	Yes	No	Decline to Respond
**If yes or decline to respond, then ask Q2. If no, then skip to Session 3.**			
25. Did you listen to the presentation for Session 2?	Yes	No	Decline to Respond
**If yes or decline to respond, then ask Q2a and Q2b. If no, then skip to Q3.**			
26. Did you listen to the presentation via Live Meeting?	Yes	No	Decline to Respond
27. Did you listen to the presentation via the archived recording?	Yes	No	Decline to Respond
28. Did you review the PowerPoint slides on the VA SharePoint site for Session 2?	Yes	No	Decline to Respond
29. Did you share the materials for this session with your staff/colleagues?	Yes	No	Decline to Respond
**If yes or decline to respond, then ask Q4a, Q4b. If no, then skip to Q5.**			
30. I shared the PowerPoint presentation.	Yes	No	Decline to Respond
31. I shared the archived recording of the session.	Yes	No	Decline to Respond

	Strongly Disagree	Disagree	Neither Agree nor Disagree	Agree	Strongly Agree	Decline to Respond
32. The information presented in this session was useful. [Insert text box so respondents can explain why or why not]	1	2	3	4	5	

33. This session improved my understanding on how the PSIs are used for hospital profiling in the private sector.						
34. This session improved my understanding on the current status of PSI use and reporting in the VA.						
35. This session improved my understanding of the VA LinKS Reports and PSI data contained in the Reports.	1	2	3	4	5	
36. After reviewing the materials for this session, I am interested in learning more about the topics presented in this session.	1	2	3	4	5	
37. After reviewing the materials for this session, I feel prepared to educate my staff/colleagues on the topics presented in this session.	1	2	3	4	5	

Session 3: How to Interpret PSI Rates (Borzecki and Shwartz)

38. Did you view or listen to any of the content for Session 3 (e.g., listened to the presentation via Live Meeting, listened to the presentation via archived recordings, reviewed the PowerPoint slide on Program's SharePoint site)?	Yes	No	Decline to Respond
**If yes or decline to respond, then ask Q2. If no, then skip to Session 4.**			
39. Did you listen to the presentation for Session 3?	Yes	No	Decline to Respond
**If yes or decline to respond, then ask Q2a and Q2b. If no, then skip to Q3.**			
40. Did you listen to the presentation via Live Meeting?	Yes	No	Decline to Respond
41. Did you listen to the presentation via the archived recording?	Yes	No	Decline to Respond

42. Did you review the PowerPoint slides on the VA SharePoint site for Session 3?	Yes	No	Decline to Respond
43. Did you share the materials for this session with your staff/colleagues?	Yes	No	Decline to Respond
**If yes or decline to respond, then ask Q4a, Q4b. If no, then skip to Q5.**			
44. I shared the PowerPoint presentation.	Yes	No	Decline to Respond
45. I shared the archived recording of the session.	Yes	No	Decline to Respond

	Strongly Disagree	Disagree	Neither Agree nor Disagree	Agree	Strongly Agree	Decline to Respond
46. The information presented in this session was useful. [Insert text box so respondents can explain why or why not]	1	2	3	4	5	
47. This session improved my ability to interpret the PSI rates on my LinKS reports.	1	2	3	4	5	
48. This session improved my ability to understand how the PSI rates in my LinKS reports are calculated.	1	2	3	4	5	
49. This session helped me better understand the strengths and limitations of the PSIs.	1	2	3	4	5	
50. After reviewing the materials for this session, I am interested in learning more about the topics presented in this session.	1	2	3	4	5	
51. After reviewing the materials for this session, I feel prepared to educate my staff/colleagues on the topics presented in this session.	1	2	3	4	5	

Session 4: How to Use the PSIs and Organizational Factors to Consider (Rivard)

52. Did you view or listen to any of the content for Session 4 (e.g., listened to the presentation via Live Meeting, listened to the presentation via archived recordings, reviewed the PowerPoint slide on Program's SharePoint site)?	Yes	No	Decline to Respond
**If yes or decline to respond, then ask Q2. If no, then skip to Session 5.**			
53. Did you listen to the presentation for Session 4?	Yes	No	Decline to Respond
**If yes or decline to respond, then ask Q2a and Q2b. If no, then skip to Q3.**			
54. Did you listen to the presentation via Live Meeting?	Yes	No	Decline to Respond
55. Did you listen to the presentation via the archived recording?	Yes	No	Decline to Respond
56. Did you review the PowerPoint slides on the VA SharePoint site for Session 4?	Yes	No	Decline to Respond
57. Did you share the materials for this session with your staff/colleagues?	Yes	No	Decline to Respond
**If yes or decline to respond, then ask Q4a, Q4b. If no, then skip to Q5.**			
58. I shared the PowerPoint presentation.	Yes	No	Decline to Respond
59. I shared the archived recording of the session.	Yes	No	Decline to Respond

	Strongly Disagree	Disagree	Neither Agree nor Disagree	Agree	Strongly Agree	Decline to Respond
60. The information presented in this session was useful. [Insert text box so respondents can explain why or why not]	1	2	3	4	5	

61. This session helped me better understand on how to use the PSIs (e.g., for benchmarking, assessing trends, case-finding).	1	2	3	4	5	
62. This session helped me better understand on where the PSIs fit among quality and patient safety measures used for quality improvement in my organization.	1	2	3	4	5	
63. This session helped me better understand on how the PSIs might be integrated into my organization's QI programs.	1	2	3	4	5	
64. This session improved my understanding on organizational factors to consider when using the PSIs.	1	2	3	4	5	
65. After reviewing the materials for this session, I am interested in learning more about the topics presented in this session.	1	2	3	4	5	
66. After reviewing the materials for this session, I feel prepared to educate my staff/colleagues on the topics presented in this session.	1	2	3	4	5	

Session 5: Using the PSIs for QI: Experiences Within and Outside the VA (Zubkoff and NCPS)

67. Did you view or listen to any of the content for Session 5 (e.g., listened to the presentation via Live Meeting, listened to the presentation via archived recordings, reviewed the PowerPoint slide on Program's SharePoint site)?	Yes	No	Decline to Respond
**If yes or decline to respond, then ask Q2. If no, then skip to Session 6.**			
68. Did you listen to the presentation for Session 5?	Yes	No	Decline to Respond
**If yes or decline to respond, then ask Q2a and Q2b. If no, then skip to Q3.**			

69. Did you listen to the presentation via Live Meeting?	Yes	No	Decline to Respond
70. Did you listen to the presentation via the archived recording?	Yes	No	Decline to Respond
71. Did you review the PowerPoint slides on the VA SharePoint site for Session 5?	Yes	No	Decline to Respond
72. Did you share the materials for this session with your staff/colleagues?	Yes	No	Decline to Respond
**If yes or decline to respond, then ask Q4a, Q4b. If no, then skip to Q5.**			
73. I shared the PowerPoint presentation.	Yes	No	Decline to Respond
74. I shared the archived recording of the session.	Yes	No	Decline to Respond

	Strongly Disagree	Disagree	Neither Agree nor Disagree	Agree	Strongly Agree	Decline to Respond
75. The information presented in this session was useful. [Insert text box so respondents can explain why or why not]	1	2	3	4	5	
76. This session made me better understand how to use the PSIs for QI.	1	2	3	4	5	
77. After reviewing the materials for this session, I am interested in learning more about the topics presented in this session.	1	2	3	4	5	
78. After reviewing the materials for this session, I feel prepared to educate my staff/colleagues on the topics presented in this session.	1	2	3	4	5	

Session 6: Wrap up call/Q&A session

79. Did you view or listen to any of the content for Session 6 (e.g., listened to the presentation via Live Meeting, listened to the presentation via archived recordings, reviewed the PowerPoint slide on Program's SharePoint site, or reviewed other materials, such as articles, web links, on the Program's SharePoint site)?	Yes	No	Decline to Respond
80. Did you listen to the presentation for Session 6?	Yes	No	Decline to Respond
**If yes or decline to respond, then ask Q2a and Q2b. If no, then skip to Q3.**			
81. Did you listen to the presentation via Live Meeting?	Yes	No	Decline to Respond
82. Did you listen to the presentation via the archived recording?	Yes	No	Decline to Respond
83. Did you review the PowerPoint slides on the VA SharePoint site for Session 6?	Yes	No	Decline to Respond
84. Did you share the materials for this session with your staff/colleagues?	Yes	No	Decline to Respond
**If yes or decline to respond, then ask Q4a, Q4b. If no, then skip to Q5.**			
85. I shared the PowerPoint presentation.	Yes	No	Decline to Respond
86. I shared the archived recording of the session.	Yes	No	Decline to Respond
87. I shared other materials related to the PSIs/PSI educational program (e.g., web links to PSI information, journal articles on the study's SharePoint site).<br>[Insert text box to explain what other materials: If you shared other materials, please explain what other materials you shared.]	Yes	No	Decline to Respond

	Strongly Disagree	Disagree	Neither Agree nor Disagree	Agree	Strongly Agree	Decline to Respond
88. The information presented in this session was useful. [Insert text box so respondents can explain why or why not]	1	2	3	4	5	
89. After reviewing the materials for this session, I am interested in learning more about the topics (e.g., costs) presented in this session.	1	2	3	4	5	
90. After reviewing the materials for this session, I feel prepared to educate my staff/colleagues on the topics (e.g., costs) presented in this session.	1	2	3	4	5	

Open ended questions:

1. What contributed most to your learning of the PSIs?
2. What contributed least to your learning of the PSIs?
3. If you felt that a particular session was especially useful, please explain.
4. If you felt that a particular session was not useful, please explain.
5. What recommendations do you have for improving the program?
6. Do you see any barriers to using the PSIs at your facility?
7. If you would like to provide us with any other comments/feedback about the PSI educational program or your opinions on the PSIs, please enter your comments/feedback below.
