# Supplementary material for: Tailoring an educational program on the AHRQ Patient Safety Indicators to meet stakeholder needs: lessons learned in the VA
Source: BMC Health Serv Res. 2018 Feb 14;18:114. doi: 10.1186/s12913-018-2904-5 (PMC5813330; doi:10.1186/s12913-018-2904-5)
Supplement: Supplementary file 4 — Informational Sheet, Interpreting the AHRQ PSIs: A Basic Overview. This file provides the informational sheet which PSI Educational Program participants could use to help them interpret and understand the PSIs. (PDF 87 kb) [file 12913_2018_2904_MOESM4_ESM.pdf]

Informational Sheet, Interpreting the AHRQ PSIs: A Basic Overview. This file provides the informational sheet which PSI Educational Program participants could use to help them interpret and understand the PSIs.

The PSIs are a set of measures that have been specifically designed to screen for potentially preventable complications occurring in the acute inpatient setting using administrative data. Although the discharge record of a particular patient stay (hospitalization) may meet the definition of a PSI, this does not necessarily mean that the event that occurred during that hospitalization was actually preventable. Only a review of the clinical record and/or an event report would likely reveal whether preventable harm to the patient actually occurred. Variation in clinician documentation of diagnoses and procedures and variation in coding practices across facilities may affect PSI rates.

This document was compiled as a basic guide to interpreting the PSIs and can be used along with the PSI Educational Program's video presentations (located on the program's SharePoint site).

Definitions

PSI rates can be reported as observed rates, observed-to-expected (O/E) ratios, and risk-adjusted rates. Observed rates are the easiest to compute and understand but do not account for patient case-mix. Therefore, facilities with a sicker-than-average patient population will be penalized in comparisons across sites, and facilities with a healthier-than-average population will appear to be performing better than they really are. Because of this, we also report O/E ratios and risk-adjusted rates, both of which adjust for patient characteristics including age, gender, Diagnosis-Related Groups (DRGs), and comorbidities as assessed by secondary diagnoses obtained during hospitalization.

Numerator

The number of cases that meet the definition for this PSI. **EXAMPLE**: The numerator for PSI 06 includes all discharges with an ICD-9-CM code for iatrogenic pneumothorax in any secondary diagnosis field.

Denominator

The number of hospitalizations at risk for this PSI. **EXAMPLE**: The denominator for PSI 06 includes all medical and surgical discharges age 18 and older defined by specific DRGs and an ICD-9-CM code for an operating room procedure. Exclude cases with ICD-9-CM codes for iatrogenic pneumothorax in the principal diagnosis field or present on admission, a diagnosis of chest trauma or plural effusion, a diaphragmatic surgery repair procedure, or any code indicating thoracic surgery, lung or pleural biopsy, cardiac surgery admissions, and obstetric admissions.

Observed Rate

This is the observed PSI rate per thousand cases. The observed rate of PSIs is calculated by dividing the numerator by the denominator and multiplying the result by 1,000.

Expected Rate of PSI events

This is the expected PSI rate per thousand given a hospital's patient case-mix.

The expected rate of PSI events is calculated by dividing the number of expected cases by the denominator and multiplying the result by 1,000. All other things being equal, certain categories of patients who are more severely ill (e.g., those with diabetes) are more likely to incur diagnoses and procedures that trigger PSIs. For two hospitals with the same denominator size, the hospital with a greater proportion of patients who are more severely ill will have a higher expected number of PSI events, and therefore a higher expected rate of PSI events, independent of its current or past observed number of PSI events.

The variables used in the AHRQ software, which generated the number of expected cases, include patient age, gender, DRGs, and comorbidities coded in hospital discharge records. The calculation of expected cases is based on a "reference population" from a national sample of non-federal hospitals.

Observed-to-Expected (O/E) Ratio

This is the ratio of a hospital's observed PSI rate to its expected PSI rate. It is calculated by dividing the hospital's observed rate by the hospital's expected rate. A lower O/E ratio (less than 1.0) is more desirable.

A ratio of 1.0 indicates that the hospital incurred the expected rate of PSI events, given its case mix.

A ratio greater than 1.0 indicates that the hospital incurred a higher-than-expected rate of PSI events. If O/E = 0.5, then the hospital had 50% fewer PSIs than were expected. For example, if 10 were expected, then 5 actually occurred.

Risk-Adjusted Rate

This is the risk-adjusted rate of PSIs per thousand patients.

Risk-adjusted rates account directly for the case-mix of a hospital's patients. The rate takes into account the "risk" of all patients at that facility. To calculate the risk-adjusted rate, the observed rates of PSIs were adjusted for age, gender, DRGs and comorbidities using the AHRQ software.

Confidence Interval (CI) Limits

The width of the CI essentially reflects the size of the sample that goes into your rate. CIs take into account uncertainty in rates so we are 95% sure the "true" rate is within the interval. Look at the CIs and risk adjusted rates when making comparisons.

PSI Composite User Technical Specification (from AHRQ)

The goal in developing composite measures was to provide a measure that could be used to monitor performance over time or across regions and populations using a method that applied at any level. Potential benefits of composite measures are to: summarize quality across multiple indicators, improve the ability to detect differences, identify important domains and drivers of quality, prioritize action for quality improvement and make current decisions about future (unknown) health care needs. Despite these advantages, composites may mask important differences and relations among indicators.

How was the PSI Composite created?

The PSI Composite is a weighted average of the risk-adjusted observed/expected (O/E) ratios of selected indicators. It is an expected value estimated from a risk-adjustment model. The weights are a combination of a "reliability" weight embedded in PSI software with user-specifications. More reliable indicators are weighted more heavily. Those indicators that are weighted have been endorsed by the National Quality Forum (NQF); indicators that were not endorsed by NQF have a weight of zero and thus, do not affect the composite calculations (see below).

The PSI Composite includes the following indicators and weights:

Indicator	Weight
PSI 03 Pressure Ulcer	0.2430
PSI 06 Iatrogenic Pneumothorax	0.0457
PSI 07 Central Venous Catheter-related Bloodstream Infections	0.1280
PSI 08 Postoperative Hip Fracture	0.0011
PSI 09 Postoperative Hemorrhage or Hematoma	0.0000
PSI 10 Postoperative Physiologic and Metabolic Derangement	0.0000
PSI 11 Postoperative Respiratory Failure	0.0000
PSI 12 Postoperative Pulmonary Embolism or Deep Vein Thrombosis	0.2360
PSI 13 Postoperative Sepsis	0.0383
PSI 14 Postoperative Wound Dehiscence	0.0124
PSI 15 Accidental Puncture or Laceration	0.2983

Additional Information:

Agency for Healthcare Research and Quality Patient Safety Indicator Homepage

Patient Safety Indicators Technical Specifications - Version 4.4, March 2012

Patient Safety Indicators (PSI) Composite Measures - Version 4.4, March 2012

AHRQ Quality Indicators™ Toolkit for Hospitals
