# Supplementary material for: Tailoring an educational program on the AHRQ Patient Safety Indicators to meet stakeholder needs: lessons learned in the VA
Source: BMC Health Serv Res. 2018 Feb 14;18:114. doi: 10.1186/s12913-018-2904-5 (PMC5813330; doi:10.1186/s12913-018-2904-5)
Supplement: Supplementary file 5 — PSI Educational Program Matrix. This file highlights information covered in each session of the PSI Educational Program and provides a list of materials referenced in each session. (PDF 213 kb) [file 12913_2018_2904_MOESM5_ESM.pdf]

PSI Educational Program Matrix. This file highlights information covered in each session of the PSI Educational Program and provides a list of materials referenced in each session.

Session #	Session Title	Objectives	Summary	Reference Materials
**1**	The AHRQ PSIs: An Overview	• Introduction to the PSI Educational Program	Provided the rationale for the PSI educational program, and a preview of each session.	None provided
**2**	The PSIs and Your Facility's Reports	• Obtain information about the importance and use of the PSIs<br>• Enhance understanding of the VA LinKS Reports and VISN-level PSI reports	PSIs provide a standardized method for identifying and measuring potential patient safety events. Facilities should still begin to familiarize themselves with the PSIs for at least 2 reasons: 1) future public reporting of PSIs; 2) examining PSI rates within a facility can help to identify improvement opportunities.	• LinKS report p. 6: PSI Composite and rates for PSI 4 (Death Among Surgical Inpatients) and 14 (Postop Wound Dehiscence)<br>• Detailed VISN report: rates of individual PSIs and PSI Composite (on IPEC SharePoint site)<br>• To request patient level data from IPEC go to this IPEC website [link].
**3**	How to Interpret PSI Rates	• Familiarize yourself with the PSI data on your reports<br>• Understand how the rates are calculated<br>• Understand how to interpret the	To review your facility's performance, look at your risk- adjusted rate. Remember also to review the 95% confidence intervals to compare your performance with other facilities, your VISN or national rates. Although administrative data quality may be limited by coding variability, the PSIs	• AHRQ PSI Technical Specifications: http://www.qualityindicators.ahrq.gov/modules/PSI_TechSpec.aspx<br>• Interpreting the AHRQ PSIs: A Basic Overview<br>• Articles on Positive Predictive Value of PSIs:<br>○ Rosen et al., Validating the PSIs, Medical Care 2012<br>○ Borzecki et al., How Valid Is the AHRQ PSI Postop Hemorrhage or Hematoma, JACS 2011<br>○ Borzecki et al., How Valid Is the AHRQ PSI Postop Physiologic and Metabolic Derangement, JACS 2011<br>○ Borzecki et al., How Valid Is the AHRQ PSI Postop

		PSI data and your reports<br>• Learn about the validity of the PSIs	can be seen as a step in the right direction because they provide a standardized way to measure patient safety. However, we suggest that you be cautious in interpreting the rates and in using them for other purposes beyond quality improvement and case finding.	Resp Failure, JACS 2011<br>○ Cevasco et al., Positive Predictive Value of the AHRQ PSI Postop Wound Dehiscence, JACS 2011<br>○ Cevasco et al., Positive Predictive Value of the AHRQ PSI Postop Sepsis: Implications for Practice and Policy, JACS 2011<br>○ Cevasco et al., Validity of the AHRQ PSI Central Venous Catheter-related Bloodstream Infection, JACS 2011<br>○ Chen et al., Detecting PSIs: How Valid is Foreign Body Left During Procedure in the VHA, JACS 2011<br>○ Kaafarani et al., Validity of Selected PSIs: Opportunities and Concerns, JACS 2011<br>• Articles on False Negatives and PSIs<br>○ Borzecki et al., Improving the Identification of Postop Wound Dehiscence Missed by the PSI Algorithm, Am J Surg 2013<br>○ Borzecki et al., Improving Identification of Postop Resp Failure Missed by the PSI Algorithm, J Med Qual 2012<br>• Articles on Processes of Care and PSIs<br>○ Borzecki et al., Is Development of PEDVT Related to Thomboprophylaxis Use, Jt Comm J Qual Patient Saf, 2012<br>○ Chen et al., Examining Processes of Care and Postop Wound Dehiscence and Accidental Puncture of Laceration, Am J Med Qual 2012

Session #	Session Title	Objectives	Summary	Reference Materials
**4**	How to Use the PSIs	• Where the PSIs fit among quality and patient safety measures used for QI in your organization<br>• How PSIs might be integrated into your organization's QI programs<br>• How the PSIs can be used for QI	PSIs are tools that can be used to drive QI, but it is best to use the PSIs in concert with other measures since they capture a narrow perspective on patient safety. When integrating the PSIs into an organization's QI program, examine how the PSIs relate to other existing patient safety measures. PSIs can be used for benchmarking against national averages, case-finding, and assessing/monitoring trends. The AHRQ Quality Indicators Toolkit is useful for integrating PSIs into QI programs.	• AHRQ Quality Indicators Toolkit: http://www.ahrq.gov/professionals/systems/hospital/qitoolkit/index.html<br>• Hart and Sweeney, Integrating PSIs into Patient Safety Programs, J Healthcare Quality 2006<br>• Rivard, Rosen, and Carroll, Enhancing Patient Safety through Organizational Learning: Are PSIs a Step in the Right Direction, HSR 2006<br>• Zrelak et al., Using the AHRQ PSIs for Targeting Nursing Quality Improvement, J Nurs Care Qual 2011
**5**	Using the PSI for Quality Improvement: Experiences in the VA	• Using the PSIs for QI activities – VA experience<br>• Using the PSIs for QI activities – Non-VA experience	Examples illustrate how the PSIs can be used for QI. The AHRQ Quality Indicators toolkit can provide guidance on integrating the PSIs into an organization.	• AHRQ Quality Indicators Quarterly Newsletter 02.04.2013

Session #	Session Title	Objectives	Summary	Reference Materials
**6**	Wrap-up and Q&A	• Highlights of each session<br>• Q&A	Questions and answers from each session addressing important topics related to the PSIs	• Zhan and Miller, Excess LOS, Charges, and Mortality, JAMA 2003<br>• Rivard et al., Using PSIs to Estimate the Impact on Potential Adverse Events on Outcomes, Med Care Research and Review 2008<br>• Carey et al., Excess Costs Attributable to Postop Complications, Med Care Research and Review 2011
